# One Health Approach to Arbovirus Control in Africa: Interests, Challenges, and Difficulties

**DOI:** 10.3390/microorganisms11061496

**Published:** 2023-06-05

**Authors:** Norvi Rigobert Bienvenu Massengo, Bachirou Tinto, Yannick Simonin

**Affiliations:** 1 Formation Doctorale de Santé et Biologie Humaine, Faculté des Sciences de la Santé, Université Marien NGOUABI, Brazzaville BP69, Congo; 2 Centre MURAZ, Institut National de Santé Publique (INSP), Bobo-Dioulasso 01, Burkina Faso; tintobachirou@yahoo.fr; 3 Pathogenesis and Control of Chronic and Emerging Infections, INSERM, University of Montpellier, Etablissement Français du Sang, 34394 Montpellier, France

**Keywords:** One Health, Africa, human health, animal health, infectious diseases, environmental health, arbovirus

## Abstract

The “One Health” concept considers that human and animal health, and ecosystems are closely related and aims to make a link between ecology and human and veterinary medicine. Due to the explosion in population growth along with the geographic and climatic conditions (equatorial and/or tropical climate), Africa is becoming a major hotspot for various socio-health issues associated with infectious diseases, including arboviruses. The incontestable advantages of a One Health approach in Africa lie in the fight against pathogens, such as arboviruses, and in the preservation of environmental, animal, and human health to ensure that the increasing high needs of this population are met as well as their protection against potential epidemics. The One Health strategy gives us a glimpse of the difficulties and challenges that the African continent faces. The importance of this approach in Africa is to establish guidelines and strategies for effective solutions and changes in behavior and harmful activities. Overall, the establishment of high-quality global health policies in the framework of the global health standards program would provide healthy and sustainable human–animal–environmental interactions for the welfare of all.

## 1. Introduction

The changes in our environment, mainly caused by human activities and the evolution of interactions between humans and animals, are, and will undoubtedly be, responsible for sanitary crises [1,2,3]. These crises manifested, in particular, through an increase in the frequency and intensity of epidemics and epizootics. Africa is becoming a major hotspot for various socio-sanitary problems concerning the health and welfare of its population due to an explosion in population growth. The problems lie in food and livestock provisions to ensure that the growing demand is met, better health provision, disease control, nutrition, reduction in space, and livestock activities. These activities lead to increased deforestation and, therefore, ecosystem disruption, with significant growth in animal abundance within shared human–animal spaces [4]. This ecosystem disruption can also lead to epidemics affecting both animals and humans. These types of epidemics show the importance of close collaboration between various specializations, from veterinary medicine to human medicine, as well as ecology, entomology, climate forecasting, and the management of societal risks [5,6,7,8,9,10].

The risk of the emergence and/or re-emergence of infectious diseases with active human-to-human transmission increases the risk of rapid and direct development of the diseases, leading to an epidemic and in some cases a pandemic. Likewise, the increase in interactions between wildlife, domestic animals, vectors for human and animal pathogens, and humans inevitably increases the risk of transmission and dissemination of zoonoses within the human population, leading to increased risk of epidemics. In addition to the challenges from the epidemics of Dengue in Burkina Faso, Ivory Coast, Nigeria, Ghana, Mali, Democratic Republic of Congo (DRC), Angola, Gabon, Sudan, and Ethiopia and the current management of the COVID-19 pandemic, there are the issues of antimicrobial resistance and environmental sanitation policies in Africa [11,12,13]. Many factors favor the strengthening of interactions between humans, animals, and the environment: increase in animal and human population movements; exploitation of natural resources; human population growth; and expansion of the human population into new geographical areas favoring the interaction between humans and wildlife. These profound modifications can have major consequences for human health [14,15,16]. At least 60% of all infectious diseases are zoonotic and account for more than two-thirds of new emerging diseases [17,18,19]. Therefore, a better understanding of the transmission of zoonotic diseases from animals to humans is an essential prerequisite for effectively anticipating and controlling zoonotic diseases.

Among these emerging diseases, the term arbovirus refers to a group of viral diseases transmitted by arthropods, such as mosquitoes, ticks, and sandflies. Vector-borne diseases are a growing burden according to the World Health Organization (WHO), sometimes causing epidemic outbreaks in various tropical regions [20]. These diseases are today, together with climate change, a risk for 80% of the world’s population. They represent 17% of the global burden of disease due to communicable diseases and cause more than 700,000 deaths per year worldwide, among which arboviruses transmitted by hematophagous arthropods (mosquitoes, ticks, and sandflies) constitute a significant threat to worldwide public health [20,21,22]. Arboviruses are mainly RNA viruses, several of which, such as DEN, Crimean–Congo hemorrhagic fever (CCHF), Zika (ZIK), West Nile (WN), Chikungunya (CHIK), and O’nyongnyong (ONN) viruses have pathogenic potential for humans, but there also many other less studied viruses that represent a potential threat to human health. The life cycle of arboviruses generally includes a sylvatic or enzootic cycle and an urban cycle which includes complex interactions between vectors, animals, and humans [23,24]. The symptoms of arboviral diseases can range from mild to severe and can include fever, headache, joint pain, rash, and muscle pain. In some cases, these diseases can lead to severe complications, such as hemorrhagic fever, encephalitis, or meningitis [24,25,26,27,28].

### 1.1. Africa Is Particularly Exposed to Arboviral Diseases

The impact of arboviral diseases in Africa is significant, particularly in low-resource settings where access to healthcare is limited [29,30]. The burden of arboviral diseases on individuals and communities can be devastating, with significant economic and social costs. Some of the most common arboviruses in Africa include Dengue) virus (DENV), yellow fever virus (YFV), Zika (ZIK) virus (ZIKV), Chikungunya (CHIK) virus (CHIKV), Rift Valley fever (RVF) virus (RVFV), and CCHF virus (CCHFV).

Arbovirosis in Africa is a complex problem that is influenced by a variety of factors, including the high prevalence of mosquito vectors that are the primary vectors. Many parts of the continent have a warm and humid climate that provides an ideal breeding ground for mosquitoes. Additionally, deforestation and urbanization create new habitats for disease-carrying mosquitoes, leading to an increase in their population [1,31]. The rapid growth of cities in Africa has led to increased population density. Urban areas often have poor sanitation and limited green spaces, creating ideal conditions for the transmission of these diseases. Many parts of Africa have a high burden of poverty, poor sanitation, and limited access to safe water and sanitation facilities, which creates favorable conditions for mosquito breeding and other arthropod vectors that transmit diseases [1,20]. This is particularly true in rural areas, where access to healthcare is often limited, and people are more likely to be exposed to mosquitoes and ticks. Moreover, mosquito control measures, such as insecticide-treated bed nets and indoor residual spraying, are not always effective or widely available in Africa. This can lead to high levels of mosquito-borne disease transmission. Poor sanitation and hygiene can also lead to the accumulation of stagnant water, which is a breeding ground for mosquitoes. In addition, inadequate waste management can attract mosquitoes and other arthropod vectors. Climate change can affect the distribution and abundance of disease vectors and the transmission of arboviral diseases [1,20,21]. Rising temperatures and changing rainfall patterns can increase the number of mosquitoes and expand the geographic range of these diseases. The movement of people and belongings across borders can facilitate the spread of arboviral diseases. This is particularly true in areas where border control and disease-surveillance measures are weak. Furthermore, arboviral diseases can spread rapidly through international travel and trade, which can introduce new strains of the virus to Africa and increase the risk of epidemics. The high prevalence of arboviral diseases in Africa is also due to the lack of effective vaccines and antiviral drugs, as well as limited access to diagnostic tests and healthcare services, particularly in rural and remote areas [29,30]. This makes the diagnosis and treatment of arboviral diseases difficult, leading to more severe disease outcomes and higher mortality rates. Moreover, there are currently no effective vaccines or specific treatments for many arboviral diseases, such as CHIK and ZIK [24]. This makes it challenging to control the spread of these diseases.

Overall, the combination of these factors makes arboviral diseases a significant economic burden to African countries, particularly in terms of healthcare costs, lost productivity, and reduced tourism revenue. Arboviruses are, unfortunately, not a priority for research and surveillance in Africa due to other health, security, and political issues. Thus, they are among the neglected diseases in many African countries, even though there is a great risk of emergence of new variants and/or re-emergence of existing variants. Many people in Africa are unaware of the risks associated with arboviral diseases and the measures they can take to protect themselves. This can lead to a lack of preparedness and a delay in seeking medical attention, which can exacerbate the impact of these diseases. The burden of these diseases is expected to increase in the future due to population growth, urbanization, and climate change, making it a major public health challenge for Africa.

### 1.2. Need for Integrated Surveillance of Emerging Viruses

The One Health approach is of great importance in the management of current and future emerging and re-emerging diseases in Africa. Tight collaboration between various actors from different disciplines is very important in the prevention, surveillance, diagnosis, and discovery of therapeutic solutions necessary for the control and/or eradication of infectious diseases. Indeed, integrated surveillance lies in the surveillance, control, and diagnosis of infectious diseases (Ebola, arbovirosis, etc.), in the research for molecules of therapeutic interest, in the training of health professionals, in connecting actors from different fields, and in establishing policies to fight against endemic and neglected zoonoses. Human behaviors and activities also show the need for this strategy. For example, deforestation by humans forces changes in wild-animal behaviors. Indeed, the animals affected have to adapt to their new environments, for example, by changing their feeding grounds and settling increasingly close to livestock breeding areas [8,31]. In turn, wild animal and other vector species of emerging or re-emerging diseases will infect domestic animals via contact with bites, food sources and/or other elements, leading to contamination chains that reach humans [4]. The balance between biodiversity and the emergence of infectious diseases increases with population density and the level of population precariousness, animal breeding behavior, deforestation, hunting, lack of socio-sanitary hygiene, and the settling in areas where infected animals are present. Human activities greatly increase the risk of disease emergence: the intensification of animal husbandry, wild-animal trafficking, deforestation (which also impacts the climate), cultural practices, conflicts leading to population movements, international travel without prior testing of those travelling (e.g., COVID-19 pandemic, Ebola, CCHF, and DEN epidemics which caused high worldwide mortality rates due to travelling of infected people). These activities create a myriad of difficulties in Africa that must be taken into account in the implementation of One-Health-type solutions.

Arboviruses are, unfortunately, not often a research or surveillance priority in Africa. In this sense, they are among the neglected diseases, although they represent an increasing risk of epidemics and the appearance of new more virulent or contagious strains. Arboviruses are a major health risk in Africa because of its climate, and the different interactions between humans, animals, and vectors. African countries must therefore adopt comprehensive policies for surveillance of and research into arboviruses.

Different methods can be used to better characterize the risk associated with arboviruses and understand their biological basis in order to better guide public health interventions. These methods include studies of biology at the environmental, vector, animal, and human level, with a trans/interdisciplinary interaction. In general, the transmission of arboviruses is influenced by a large number of factors related to the biology of hosts, vectors, and viruses, which are inter-related within dynamic natural environments and influenced by abiotic factors, such as climate or hydrography [32]. A comprehensive understanding of the full range of factors contributing to the infection of accidental hosts, which may be distributed across diverse environments with varying levels of risk, usually requires the joint analysis of multiple sources and types of data analyzed at different scales. Obtaining an exhaustive epidemiological portrait of arboviruses in a given region therefore represents a considerable challenge, particularly in the context of emergence and/or re-emergence, especially since climate changes could lead to the emergence of these viruses in areas previously spared from these health crises.

**Figure 1:**
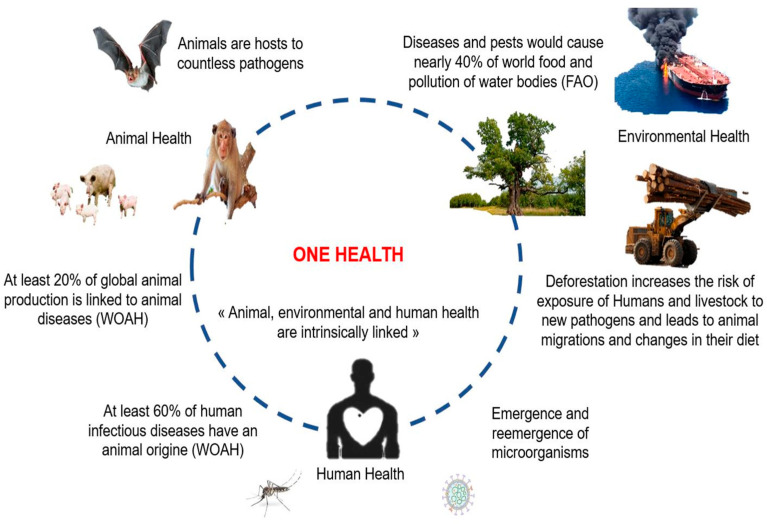
Schematic representation of the interrelatedness of animal, environmental, and human factors justifying a One Health approach: impact of environmental and animal health on human health, and, conversely, the impact of human behaviors and activities on the emergence and re-emergence of micro-organisms. (Adapted from WOAH: World Organization for Animal Health; FAO: Food and Agriculture Organization).

Consequently, the need to understand the emergence of a disease in humans through an approach that integrates a large number of environmental parameters, described as global, has been reinforced [33,34]. Hence, the concept of “One Health” was developed by basing the study of these issues on multidisciplinary and multisectoral approaches. One Health strategies involve studying the interactions between animals, humans, and their various environments [33]. Instead of the traditional approach of studying the circulation of a pathogen—for example, by focusing on the appearance of symptoms exclusively in human cases—One Health encourages collaboration and synergies between all sectors and actors with fields of activity that can impact human and animal health [33]. The principle is therefore to break away from a monothematic and anthropocentric epidemiological vision of health issues [35]. Ultimately, the objective of One Health is to participate in the mitigation and prevention of health risks related to animal–human–ecosystem interactions by strengthening the international capacity to anticipate and detect infectious diseases and provide concrete response strategies (Figure 1).

Tight collaboration between animal, environmental, and human health institutions is important to find solutions and effective control approaches at both local and international levels. These efforts must include Africa, especially given its climate, rapid population growth, and slow economic growth, making Africa at risk for the emergence and/or re-emergence of epidemics. For the moment, no African country has taken the lead for the continent, but instead, individual countries are organizing themselves for carrying out studies with a One Health aim, sometimes with local funds and very often with foreign funds. This is particularly the case today for common North–South diseases (the Global Health approach) [36,37]. The goal of North–South technology transfer is to perform studies investigating better problem characterization and to find adequate solutions [38,39,40,41,42,43]. West Africa has made progress in this area as evidenced by the handful of studies published over the last 10 years [38,39,44,45,46]. On the other hand, Central Africa is lagging behind. This is due to the favorable weather climate for the emergence or the re-emergence of these diseases.

The effectiveness of the One Health approach has been demonstrated with zoonoses responsible for emerging and/or re-emerging diseases linked to epidemics in certain regions of Africa (Kenya, DRC, Uganda, Burkina Faso, Nigeria, etc.) [39,40,42,43]. Even if awareness is applied to human health, not taking into account animal and/or environmental health is likely to maintain these diseases and, therefore, lead to their re-emergence [31,33,47]. The perspective here is to highlight the importance of integrated strategies and to highlight the issues related to the implementation of these approaches in Africa.

### 1.3. Methodology

**Figure 2:**
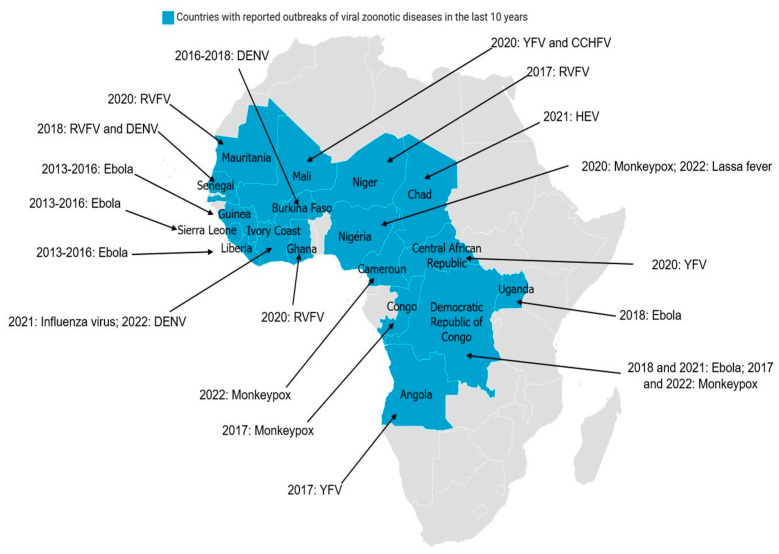
Distribution of major viral zoonotic diseases in Africa over the last 10 years: Blue represents countries with reported outbreaks of viral zoonotic diseases in the last 10 years. The virus name and years of diagnosis are indicated. (CCHFV: Crimean–Congo hemorrhagic fever virus; DENV: dengue virus; RVFV: Rift Valley fever virus; HEV: hepatitis E virus; YFV: yellow fever virus.

**Figure 3:**
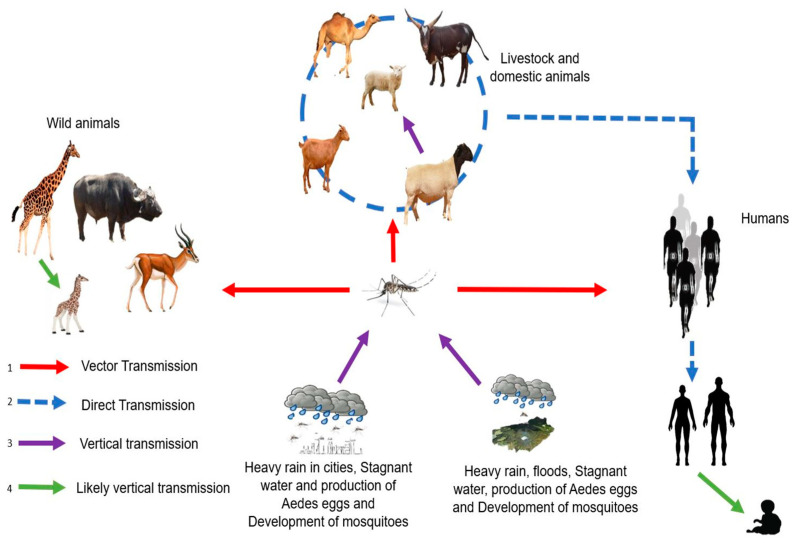
Transmission cycle of RVFV with different modes of transmission: (1) Humans, livestock, domestic and wild animals are infected through mosquito bites (red line). (2) Direct transmission between humans and domestic animals (exposure to body fluids, blood, and tissues of infected animals), between domestic animals and between humans (blood transfusion) (blue dotted line). (3) Vertical transmission in vectors and domestic animals (purple line). (4) Likely vertical transmission in wild animals and humans (green line).

**Figure 4:**
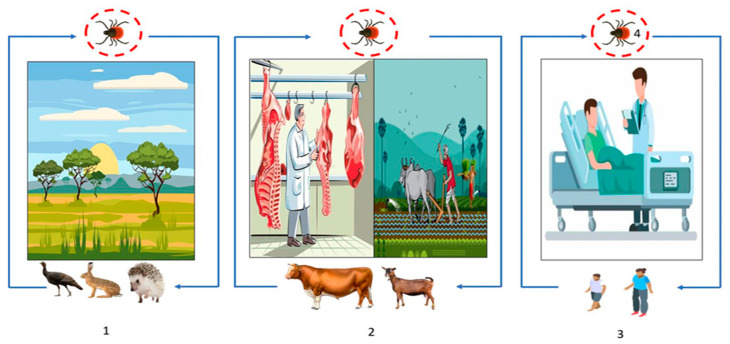
Transmission cycle of CCHFV: (**1**) Wild animal tick–host interactions, affecting, or not, animal health (blue line) and (**2**) animal farming (blue line), (**2**) and (**3**). The overlap of human health with animal health through the routes of virus transmission, most commonly through tick bites or tick crushing, and secondarily through contact with infected animal blood during slaughter or during contact with animals in livestock and agricultural areas (blue line). (**3**) Human-to-human transmission can also occur in a domestic or nosocomial infection when no appropriate personal protective equipment is used (blue line). (**4**) Transstadial and transovarian transmission between ticks is possible (red circle).

**Figure 5:**
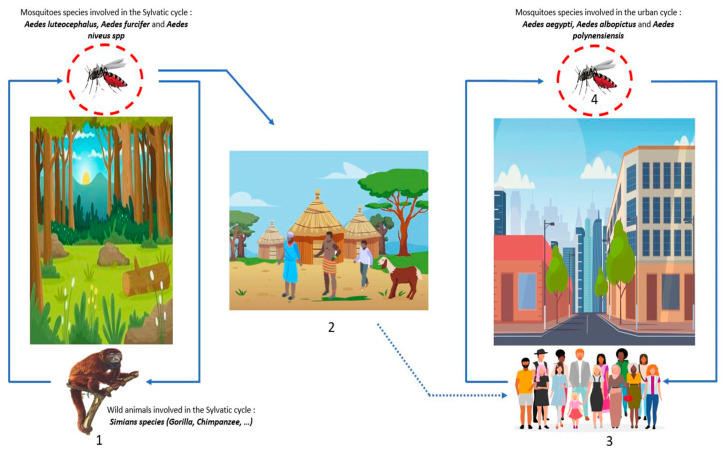
DENV transmission cycles: (**1**) Wild forest animals are infected by the bites of mosquito-*Aedes* (blue line), this cycle is the sylvatic cycle. (**2**) The dwellings near the forests or within the sylvatic cycle are named the zones of emergence and/or re-emergence of the virus. The movement of populations infected by mosquito bites to urban areas leads to the urban cycle. (**3**) The urban cycle with transmission to urban mosquitoes, then transmission to humans induced by infected urban mosquitoes (blue line). (**4**) Transovarian transmission of infected mosquitoes is possible both in the forest and in urban areas (red circle).

We analyzed several reports on the One Health concept and highlight the relevance of using this approach in the control of arboviruses in Africa, while at the same time addressing the challenges and difficulties related to its implementation. A systematic literature search related to the topic was first performed in electronic databases (PubMed, Scopus, Google scholar, Wiley online library). We also searched data on the WHO and the Institute of Research for Development (IRD) websites. All collected data were then used to discuss: (1) the relevance of the One Health concept in the control of arboviruses in Africa and (2) the challenges and difficulties of implementing this concept on the African continent. The search included research articles (in English and French) and news articles published between January 2009 and April 2023 (that last 14 years). A total of 863 articles were reviewed and all articles identified as irrelevant were excluded. Exclusion criteria included type of study, language of publication, nature of the pathogens addressed, and incomplete technical and/or clinical data. The articles were analyzed first on the basis of the title, then the abstract, and then, finally, the full text. A total of 71 articles were included for study. We combined the following search terms: “One Health and Africa” and “human health” or “environmental health” or “animal health” or “arbovirus and emerging diseases” or “re-emerging diseases” and “Africa climate”. Figure 2 was generated using the online software Datawrapper and Figures 1, 3–5 were generated using PowerPoint 2019 software.

## 2. Key Examples of Arboviruses Requiring One-Health-Type Surveillance in Africa

In regard of various epidemics in Africa (e.g., RVF, YF, DEN, CCHF, Ebola, and avian influenza), the health authorities of certain countries have thought of a multidisciplinary strategy to fight against these epidemics [27,48,49,50]. The creation of technical control committees in various countries between 2000 and 2006 has made it possible, over time, to strengthen the partnerships between agencies of the various governments in order to test multidisciplinary approaches. From 2005 to 2011, shared programs were set up between different countries to deal with epidemic and pandemic threats (e.g., the creation of One Health Central and Eastern Africa (OHCEA)). This is a network bringing together different African countries in training, research, and approaches to One-Health-type solutions. Several other institutions are also emerging in Africa with the intention of providing solutions to the challenges related to the implementation of the One Health strategy [38,51,52]. Epidemics of viral zoonotic diseases, reported in several African countries during the last 10 years (Figure 2) have shown the importance of the One health concept in pathogen surveillance and epidemic management in Africa. The tight collaboration between countries and the sharing of common experiences are crucial.

The relationship between a pathogen (virus, bacteria, parasite, etc.), and its host is rather dynamic and the environment plays also an important role in the nature and dynamics of an epidemic [53]. This makes the surveillance of many emerging pathogens complex.

### 2.1. Rift Valley Fever (RVF)

RVFV is an arbovirus caused by a *Phlebovirus* of the *Phenuiviridae* family. RVF is endemic in Africa and affects domestic ruminants, such as cattle, sheep, goats, and camels [14]. RVF can spread to humans and has been responsible for former epidemics. RVF infection can result in the need for abortion among pregnant females and high mortality in young animals. Transmission of the virus is ensured by *Aedes*-type mosquitoes that transmit the virus during blood meals both in animals (domestic or wild) and humans, with contact between infected animals also allowing for virus spread (Figure 3). In humans, RVFV infection causes influenza-like illness that may progress to more serious complications, such as meningitis encephalitis, retinal lesions, anemia, and hemorrhagic syndromes [14].

From 1997 to 2016, several outbreaks were observed in Kenya, Somalia, Tanzania, Mauritania, Senegal, Algeria, Morocco, Tunisia, and Niger [10,14,27]. A viral emergence in a given territory takes place in three main stages: viral introduction, viral establishment, and then viral spread. In the case of RVF, several factors intervene and play a crucial role in the outbreak of epidemics/epizootics. These factors include: the diversity and abundance of the vector species as well as their mammalian hosts (domestic and wild) and the degree of immunity against RVF and the environmental conditions (temperature, rainfall, vegetation). These epidemics were influenced by several factors acting concomitantly: heavy rainfall increasing the development of competent mosquito species, animal movements, and a low level of host immunity against RVF. Ruminant farms near human populations also favored the outbreak.

The lack of diagnostic tools for this disease remains a major problem. In light of this, the development of One-Health-type approaches aims to make it possible to detect cases and establish strategies for limiting cases of both human and animal infections, and avoid environmental dissemination by contact between wild and domestic fauna, in a so-called global control plan. A large part of the African continent has an equatorial and/or tropical climate. This climate is characterized by heavy rainfall, with almost daily rainfall in certain regions (the majority being nocturnal (equatorial forest zone)) and annual rainfall totals over 150 mm [31,47]. These recurrent precipitations favor the accumulation of pools favorable to the growth of mosquito vectors of RVF [54]. Here, the One Health strategy is important as it denotes the population distribution of vectors present in defined areas, thanks in particular to entomological studies. Knowledge of vector species in given areas makes it possible to identify the pathogens that can be transmitted, and provide effective control strategies against these vector species to avoid the inter-species and human transmissions causing potential epidemics. From this same approach, parallel studies must be carried out in both ruminants and humans exposed to potentially infected ruminants to limit the spread. Overall, the One Health approach in the context of RVF must challenge all axes through close trans/interdisciplinary collaboration, including vector, environmental, animal, and human control.

### 2.2. Crimean–Congo Hemorrhagic Fever (CCHF)

CCHF is a viral hemorrhagic fever caused by a virus from the family *Nairoviridae*, genus *Orthonairovirus*. It is a zoonosis that also has a high rate of human-to-human transmission and produces epidemic outbreaks in humans [26,55,56]. The clinical picture of the disease includes three phases: (i) pre-hemorrhagic phase that lasts on average 5 days during which patients present a high fever accompanied by myalgias, headaches, retro-orbital pain, gastrointestinal symptoms (nausea/vomiting and diarrhea), and, sometimes, neck stiffness; (ii) a hemorrhagic phase characterized by bleeding from mucosal tissues (epistaxis, hematemesis, hemoptysis, and hematuria) and sometimes bleeding from the skin (ecchymosis/purpura); and (iii) convalescence marked by fatigue, tachycardia with unstable blood pressure, temporary alopecia, and memory disorders [28]. The mortality rate is approximately 40% [55,57]. The mode of transmission is most often by inoculation of the virus during tick-feeding (hares, birds, rodents, pets, humans). Exposure to contaminated blood or secretions can also cause the disease, with the slaughter of infected animals also favoring the transmission of the virus. CCHFV life initially involves silent transmission between multiple vertebrate hosts (wild and/or domestic) with the potential absence of overt clinical disease in hosts and ticks (Figure 4). In the tick vector, trans-ovarian and trans-stadial transmission of the virus is noted [58]. Farmers, slaughterhouse employees, or veterinarians are the major population at risk. Detection of previous infection is made by an enzyme immunoassay (ELISA) by detection of IgG and IgM antibodies, and confirmation requires reverse transcriptase polymerase chain reaction (RT-PCR). Virus isolation can also be done by cell culture. However, a BSL-4 laboratory and well-trained personnel are required due to the pathogenicity of the virus. Vaccines exist but remain ineffective due to the diversity of viral strains in certain areas of the world [59].

Considered the second most common arbovirus in the world due to its geographical distribution, CCHFV is endemic in several African countries, such as DRC, Nigeria, Namibia, Mali, Burkina Faso, Central Africa Republic, Senegal, Uganda, South Soudan, Ethiopia, Tanzania, Madagascar, Mauritania, South Africa, Kenya, and Senegal, as well as in Asia (Iran, Iraq, Kazakhstan, Uzbekistan, Pakistan, China, etc.), South-Eastern Europe, and the Middle East (Russia, Ukraine, Croatia, Macedonia, etc.) [26,60,61,62]. Potential reasons for the emergence or re-emergence of CCHF include anthropogenic factors, such as changes in agricultural activities.

Due to this very complex cycle, in which hosts and vectors are themselves strongly influenced by environmental parameters, a One Health approach is required for the prevention and control of this disease [63]. The integrated surveillance of CCHF can help to identify potential sources of infection, track the spread of the disease, and inform prevention and control measures to reduce the risk of transmission to both humans and animals. This approach requires the correct identification (diagnosis) of human cases and identification of tick vectors and animal reservoirs to better understand the risk factors and prevent the spread of the disease. Surveillance data from both animal and human populations can provide valuable information on the incidence, prevalence, and distribution of the disease. The animal health component of CCHF surveillance would involve surveillance for the presence of the virus in domestic and wild animal populations, including livestock and wild game. This would involve regular testing of blood and tissue samples from animals, as well as surveillance of tick populations in the area. The human health surveillance would involve surveillance for cases of the disease in humans. This would involve active surveillance in healthcare facilities and communities, as well as of travelers and migrants who may have been exposed to the virus in endemic areas. The environmental health surveillance would involve surveillance for the presence of ticks and other potential vectors in the environment. This would involve study of tick populations and their habitats, as well as the monitoring of environmental factors that may contribute to the spread of the disease. Surveillance at the tick, animal, and human levels is necessary for effective and continuous control of CCHF. Moreover, the difficulty in identifying symptoms in a population of sick animals makes it difficult to cull or quarantine animals to prevent the spread to other animals or to humans. Thus, controlling animal movements and all human–animal interactions can also further reduce the number of cases.

In Africa, improvement in technical platforms for the detection of CCHF is required. Indeed, the 451 antigenic cross-reactivities with nairoviruses prevents serological analyses due to the high rate of false positives. In this sense the One Health approach will make it possible to pool funds and direct funding towards the most appropriate strategies for all, and an appropriate screening strategy [28]. Although baseline detection of the virus in ticks is most important, many other factors may determine the long-term maintenance and persistence of the virus in ticks over time, including rates of transstadial and transovarial transmission, variety of food contamination, and relative densities of ticks and animal hosts [55]. To better understand the frequency and contribution of transstadial/transovarial transmission on virus maintenance in tick populations, surveillance efforts should focus on collecting questing ticks, although this may be much more difficult than collecting ticks from animal hosts. Physical, chemical, and biological control of human ticks are robust approaches that will significantly reduce the tick vector population.

The One Health approach to CCHF surveillance also involves promoting awareness and education about the disease, particularly in areas where CCHFV is endemic. This includes educating communities about the importance of tick control, proper animal husbandry, and safe food-handling practices.

### 2.3. Dengue (DEN)

DENV belongs to the *Flaviviridae* family, genus *Flavivirus*. There are four serotypes of DENV: DENV-1, DENV-2, DENV-3, and DENV-4 [64]. DENV is the most widespread arbovirus in the world with approximately 390 million cases each year, of which 25% present clinical manifestations, with more than 25 thousand annual deaths [64,65]. DENV is transmitted via mosquito bites during the blood meal, the mosquito responsible for this transmission belongs to genus *Aedes*. Like all arboviruses, transmission is either sylvatic, where the virus circulates between wild animals, such as Simian species (gorilla, chimpanzee, etc.), and mosquitoes (*Aedes luteocephalus*, *furcifer,* and *niveus* spp.) without human contact; or urban, where the virus circulates between mosquitoes (*Aedes aegypti*, *polynensiensis,* and *albopictus*) and humans through constant contact with the bite occurring preferentially during the day (Figure 5). Infected mosquitoes infect vertebrate hosts during meals, and, conversely, uninfected mosquitoes become infected during blood meals in hosts with high viremia [25,66]. Other modes of transmission include blood transmission, accidental exposure to biological fluids, and vertical transmission (mother–child), that is most often detected through breastfeeding, ranging from asymptomatic forms to severe forms (hemorrhagic forms) [25,67]. The symptoms are most often: headache; myalgia; arthralgia; asthenia, which can lead to nausea and vomiting, in the most severe cases; neurological, and digestive; and visceral damage can be observed [64]. The febrile state is most frequent during a DENV infection, but others symptoms such as headache, myalgia, arthralgia, and asthenia are observed, which greatly complicates the diagnosis as it can be confused with other diseases (malaria, typhoid fever, etc.).

DENV is endemic in most tropical and subtropical regions [64]. This virus has spread rapidly throughout the world and currently over half of the world’s population lives in areas at risk. Currently, Asia remains the most affected continent, followed by America. Africa has not been spared given the presence of mosquitoes of the genus *Aedes*; vectors of DENV. The lack of quality diagnostic tools in Africa makes diagnosis difficult and prevents knowledge of the disease. From 1927 to 2022, several DEN epidemics were declared in Africa, particularly in South Africa, Senegal, Burkina Faso, Mali, Nigeria, Ghana, Ivory Coast, DRC, Angola, Gabon, Sudan, and Ethiopia [13,64,68]. Diagnosis of DEN in African countries is far from comprehensive, partly due to the lack of trained personnel and appropriate equipment, as this virus can cross-react with other flaviviruses.

Prevention and control efforts are crucial to reduce the impact of the disease on humans. The interest in DEN surveillance by the One Health approach lies in its ability to provide a more comprehensive understanding of the disease and its transmission dynamics. Dengue involves multiple hosts and vectors, including humans, mosquitoes, and non-human primates, although dengue epidemics are currently linked to an urban transmission cycle. A One Health approach to DEN surveillance can provide a comprehensive understanding of the disease and help in developing effective prevention and control strategies. Surveillance of DENV circulation in mosquitoes (mainly *aegypti* and *albopictus*) helps in the early detection of cases and outbreaks, and facilitates a rapid response to control the spread of the disease. In addition, early detection of circulating DENV serotypes could identify potential new emerging strains. Then, by integrating data from human, animal, and environmental sources, public health officials may identify potential outbreaks early and take proactive measures to prevent their spread. This approach can lead to the development of more effective and sustainable strategies for disease prevention and control, as well as contribute to the overall health and well-being of communities.

## 3. Challenges and Difficulties of One Health Approaches in Africa

Although the One Health concept applies concretely to certain studies and certain pathogens, many African countries encounter practical difficulties in implementing approaches, particularly in terms of communication between actors in the field (stockbreeders, farmers, physicians, investigator, etc.). Most African countries are poor and located in areas at high risk of disease emergence due to their climate, proximity to risk areas, and human activities. Therefore, the pooling of funds and One-Health-type policies would be very useful, both in the reduction of unnecessary expenditure and in the establishment of priority policies related to the surveillance and control of emerging or re-emerging diseases [4]. The challenges in Africa lie in the application of trans/interdisciplinary strategies, the creation of surveillance and local control posts, mobile laboratories for screening cases, establishing maps of human population exposure to wildlife, creating maps of the distribution of disease vector species and associated diseases, regulating the consumption of high-risk foods, and establishing compliant breeding policies thanks to a network for animal-breeding-site surveillance. Many African veterinary and medical institutions are still in the development phase and so collaboration between them is not well established. Very often the goodwill of the various partners involved is just not adequate given the harsh realities behind the planning, execution, and budgeting specific to the national and regional context of each country. Moreover, the sociological and cultural dimensions specific to each region in order to mobilize the local communities should be considered. These are essentially linked to the monitoring and control of emerging diseases, but also of certain endemic diseases such as some arbovirosis and malaria, which are indeed already circulating in many African countries.

Analysis of the flaws in the management of the COVID-19 crisis has shown that the One Health strategy is indeed rarely used in many countries [69]. The strategy often lacks structured and operational management, as well as political support. Joint action requires not only enhanced communication around risk analysis and prevention, but also active collaboration during the management of the health crisis. The initial diagnostic difficulties in many countries during the COVID-19 pandemic also highlighted the lack of transferability between veterinary and human diagnoses, with many obstacles existing at both the cultural and logistical levels [69]. In Africa, the development of diagnostic tools is a challenge. For example, during the Ebola epidemic a substantial amount of time elapsed between the index case and the identification of the virus, and then a relatively long time elapsed until the implementation of emergency diagnostic policies led to the discovery of the disease-causing pathogen. Even today, It is still difficult to detect arboviruses in several regions of Africa due to the lack of an adequate technical platform [6,65]. This has a strong impact on the time it takes to implement response policies. Hence, not being able to identify the pathogen means it is difficult to know which response strategy is the most suitable to fight the disease.

An integrated health strategy has indeed operational consequences that disrupt the existing methods of study, surveillance, prevention, and action—giving rise to resistance to implementation. Implementation of One Health requires a change in the understanding of risks and practices [70]. The various existing networks sometimes show insufficiencies in coordination and strategic management. Although the One Health approach has made considerable progress at the institutional level, its operational capacity is still limited and focuses, above all, on the control of infectious diseases, such as CCHF or RVF, rather than on the overall notion of health promotion and of resilience through sustainable practices such as “strengthening health systems” [71]. We need to coordinate the implementation of this operational field research at the highest level of health and political governance. Very often, the programs in place are faced with policies, structures, and funding that are not well suited to transdisciplinary approaches, working in sectors that are still too compartmentalized and often with a short time frame (a few years), imposing a short-term vision on programs that, conversely, require sustainability. Operating on One Health interfaces requires adaptation of health governance mechanisms at global, national, and local scales with a wide variety of institutions and actors with very different backgrounds, priorities, sources, and levels of funding. Gaps and weaknesses in veterinary, entomological, and human health services are numerous. This is particularly the case in the most rural or forested areas, where the interface between humans, animals, and vectors is, nevertheless, very strong. The low support from the populations for the proposed approach, the lack of qualified human resources, as well as of suitable financing or infrastructure, often constitute very difficult obstacles to overcome.

The centralization of data is important for an overall quality analysis based on the economic and geographical networks in place. It is also important to establish shared regulations between the different sectors involved, standardized operating protocols, effective control instruments, the creation of health structures for rapid treatment, and laboratories sufficiently equipped to maintain health and surveillance systems during crises. It is also important to improve staff training in One Health strategies by introducing the importance of this notion into the training paths of students from the various disciplines concerned.

## 4. Conclusions

The recent outbreaks of arboviruses (DENV, RVFV, CCHFV, etc.), and the COVID-19 pandemic have demonstrated the need for an integrated health strategy and have encouraged us to plan for the prevention and management of future crises. Africa is particularly exposed to the risks of the emergence of new pathogens given its environment of high demographic growth with a favorable climate to emerging/re-emerging diseases. A special effort must be made in Africa to ensure a trans/interdisciplinary approach for safer health and more effective management of future epidemics. This management involves coordinated multi-sector actions at different scales, from local to international. The One Health concept should be considered as an approach that goes far beyond the prevention of health crises; it is indeed a holistic vision of health that considers the close links between health, environment, climate, food, and agriculture. Implementing the One Health concept requires political commitment and adherence to a number of core principles, including socio-political and multi-cultural parity, inclusion, and community involvement, defined by the One Health high-level interdisciplinary expert group.

## Data Availability

Not applicable.
